# Extracellular Matrix Stiffness Enhancement Promotes Docetaxel Resistance in Prostate Cancer via Inhibition of Apoptosis Mediated by Upregulation of PRRX1

**DOI:** 10.7150/ijms.111171

**Published:** 2025-07-25

**Authors:** Jiahao Chen, Mengting Chen, Zhiwen Xie, Luheng Shen, Juntao Jiang, Shujie Xia

**Affiliations:** 1 Department of Urology, Shanghai General Hospital, Shanghai Jiao Tong University School of Medicine, Shanghai 200080, China.; 2 Department of Critical Care Medicine, Shanghai General Hospital, Shanghai Jiaotong University School of Medicine, Shanghai 200080, China.

**Keywords:** ECM stiffness, PCa, docetaxel, drug resistance

## Abstract

**Background:** Prostate cancer (PCa) poses a significant health burden for men, with docetaxel constituting the primary therapeutic option for patients with metastatic PCa. However, the mechanisms governing docetaxel resistance remain incompletely understood. Several studies have implicated the role of the extracellular matrix (ECM) stiffness in cancer drug resistance, yet the precise role of ECM stiffness in docetaxel resistance in PCa remains elusive. The aim of this study was to explore the influence of ECM stiffness on docetaxel resistance in PCa and elucidate the underlying molecular mechanisms, thereby providing novel insights into PCa treatment.

**Methods:** Polyacrylamide gels of varying stiffness were utilized to mimic different ECM stiffness conditions. The sensitivity of PCa cells to docetaxel was evaluated using CCK-8, TUNEL staining, flow cytometry, and western blotting. RNA-seq was employed to analyze the transcriptomic effects of different ECM stiffness on PC-3 cells. Western blotting, qPCR, and siRNA were utilized to validate the regulatory role of the key gene in the sensitivity of PCa cells to docetaxel under varying stiffness conditions.

**Results:** Our findings indicate that high ECM stiffness enhances docetaxel resistance in PCa cells by inhibiting docetaxel-induced apoptosis. This process is mediated through the integrin-related mechanotransduction pathway. Specifically, high ECM stiffness upregulates the expression of PRRX1, thereby promoting docetaxel resistance in PCa cells.

**Conclusions:** High ECM stiffness promotes docetaxel resistance in PCa, with PRRX1 identified as a pivotal gene in this process. These findings contribute to a deeper understanding of the mechanisms underlying docetaxel resistance in PCa and may inform the development of novel therapeutic strategies.

## Introduction

Prostate cancer (PCa) continues to pose a significant threat to men's health^1^. Currently, a multitude of pharmacological and non-pharmacological interventions are available for managing PCa, significantly extending patient survival. Among these, androgen deprivation therapy (ADT) stands as a cornerstone treatment^2^. However, after an average duration of 12 to 18 months, most patients develop resistance to ADT, progressing to castration resistant prostate cancer (CRPC)^3^. Docetaxel has emerged as the primary therapeutic option for CRPC patients, with the TAX327 study demonstrating its survival benefit over mitoxantrone^4^. Additionally, the CHAARTED trial further established that combining docetaxel with ADT as a first-line treatment for metastatic hormone-sensitive PCa offers a notable survival advantage compared to ADT alone^5^. Nevertheless, long-term therapy and genetic variations continue to pose challenges through the development of drug resistance.

The intricate mechanisms underlying docetaxel resistance remain incompletely understood. Known contributors to this resistance encompass alterations in microtubules, modulation of drug transporter protein expression, perturbations in cell cycle-related proteins and cell cycle progression, as well as changes in chemokines and signal transduction proteins^6^, ^7^. Notably, variations in β-microtubule protein isoforms, particularly classes III and IV, correlate with docetaxel efficacy^8^. The ABC transporter protein superfamily, which includes members such as ABCB1, ABCC1, and ABCC4, facilitates docetaxel efflux, thereby mediating resistance in PCa^9^. Furthermore, apoptosis-related protein alterations also play a pivotal role, with BCL2 family inhibition sensitizing PCa to docetaxel^10^. Recently, the role of the ECM in docetaxel resistance has garnered increasing attention^11^.

The extracellular matrix (ECM), composed of macromolecules like collagen, elastin, proteoglycans, and glycoproteins^12^, functions as a structural scaffold maintaining tissue and organ homeostasis^13^. It also constitutes a vital component of the tumor microenvironment, influencing tumorigenesis and progression^14^. Studies have increasingly highlighted the ECM's capacity to promote tumor growth and metastasis through interactions with tumor or mesenchymal stromal cells, initiating various signaling pathways^15^. Besides macromolecular composition, mechanical properties such as stiffness, stress, tension, and porosity also mediate the ECM's role in cancer.

The impact of ECM stiffness on tumor drug resistance is a multifaceted process. For instance, in breast cancer, increased ECM stiffness upregulates multidrug resistance protein 1, enhancing drug efflux^16^. Similarly, high ECM stiffness preserves tumor stem cell properties, fostering drug resistance^17^. A general trend towards drug resistance with increasing ECM stiffness is observed in multiple tumor types. In pancreatic cancer, high ECM stiffness induces epithelial-mesenchymal transition (EMT), reducing paclitaxel sensitivity^18^. In hepatocellular carcinoma, stiff substrates diminish cisplatin-induced apoptosis in tumor cells compared to soft substrates^19^. Conversely, lower stiffness stimulates the expression of ABC transporter proteins (ABCB1 and ABCB4) in ovarian cancer cells, leading to cisplatin and paclitaxel resistance^20^. This variability underscores cancer cells' plasticity in adapting to different ECM stiffness levels. However, the specific influence of ECM stiffness on PCa's sensitivity to docetaxel remains elusive, necessitating further investigation.

Integrin is a critical mechanosignal transducer that mediates the tumor-promoting effects of ECM stiffening. For example, substrate stiffening can promote anabolic metabolism through integrin^21^. Integrins also play an important role in the progression of prostate cancer^22^. Integrin suppresses prostate cancer metastasis via regulation of the Hippo pathway and enhancement of collagen I binding^23^, ^24^. Emerging evidence highlights the contributory role of integrins in tumor drug resistance development^25^, ^26^, underscoring the necessity of investigating their potential involvement in the aforementioned mechanism.

This study aims to elucidate the role of ECM stiffness in docetaxel resistance in PCa through *in vitro* assays. By revealing the molecular mechanisms by which ECM stiffness modulates PCa's sensitivity to docetaxel, this research endeavors to pave the way for novel strategies to overcome PCa chemoresistance.

## Materials and Methods

### Cell culture

Human PCa cell lines PC-3, DU145 and C4-2B were obtained from the Cell Bank of the Chinese Academy of Sciences (Shanghai, China). These human PCa cells were grown in 1640 medium (Gibco, USA) containing 10% fetal bovine serum (FBS, Gibco, USA) and incubated at 37 °C with 5% CO2.

### Preparation of polyacrylamide gel

Polyacrylamide gel with different stiffness was prepared by changing the ratio of acrylamide and bisacrylamide^27^. APS (Aladdin, Shanghai, China) in a volume of 1/100 of the total volume and TEMED (Aladdin, Shanghai, China) in a volume of 1/1000 of the total volume were added to the mixed solution to promote polymerization. Sulfo-SANPAH (Sigma, USA) and collagen type I (Corning, USA) were added dropwise on the gel surface and irradiated with UV light for 30 min.

### Phalloidin staining

Cells were fixed with 4% paraformaldehyde and treated with 0.5% Triton X-100 for 5 min. After washing with PBS, the cells were incubated with phalloidin staining reagent (Thermo, USA) for 40 min, protected from light. After washing again with PBS, the nuclei were restained dropwise with DAP. Cells were imaged with a fluorescence microscope (Leica Microsystems, Heidelberg GmbH, Germany).

### Cell viability

Cells were plated in 96-well plates and were treated with different concentrations of docetaxel for 24 or 48 h. Cell Counting Kit-8 reagent (CCK-8, Ncmbio, China) was then added and cell viability was quantified by measuring the absorbance at 450 nM. The median inhibitory concentration (IC50) was calculated using GraphPad Prism 9 (GraphPad Prism 9.0.0, USA).

### TUNEL staining

TUNEL staining was performed using a TUNEL Apoptosis Assay Kit (Beyotime, China). Cells were fixed with 4% paraformaldehyde and treated with 0.5% Triton X-100 for 5 min. After washing with PBS, TdT enzyme solution was added dropwise and incubated at 37°C for 1 h. Cells were imaged with a fluorescence microscope (Leica Microsystems, Heidelberg GmbH, Germany).

### Flow cytometry analysis

Flow cytometry analysis was conducted to assess cell apoptosis. The cells were detached using trypsin and collected by washing and centrifugation with cold PBS. Cell apoptosis was examined using the Annexin V-FITC/PI apoptosis detection kit (Thermo, USA) as per the manufacturer's instructions. The pre-treated cells were filtered, and the fluorescence of FITC/PI was measured using a Accuri C6 flow cytometer (Becton, Dickinson and Company, USA). Data were analyzed using FlowJo V10.0 software (FlowJo, USA).

### Western blotting assay

Total protein was isolated using RIPA buffer (Beyotime, China). Proteins were subjected to SDS-PAGE, transferred to PVDF membranes and incubated with primary and secondary antibodies as previously described. Detection was performed with ECL reagent (Ncmbio, China). All experiments were performed at least three times.

### RT-qPCR

Total RNA was extracted using Trizol reagent following the manufacturer's manual. RNA (1 μg) was then reverse transcribed into cDNA by HyperScript III RT SuperMix. To quantify gene expression levels, the relative quantification values for mRNA were calculated by the 2^-ΔΔCt^ method using GAPDH as the internal reference. All relevant reagents were purchased from NovaBio (NovaBio, China) and all experiments were performed three independent times.

### RNA-seq and data analysis

PC-3 cells cultured on 0.1kPa and 4kPa substrates for 24 hours were collected in three replicates each. Total RNA of these 6 samples were extracted and sequenced using IlluminaNovaSeq6000 by Majorbio Company (Shanghai, China).

Raw reads were pre-processed by Majorbio using their in-house pipeline. Differentially expressed transcripts were identified as those with |log2(fold change) | ≥1 and corrected p value <0.05.

### Sirna transfection

For siRNA transfection, PC-3 cells were cultured on 0.1kPa and 4kPa substrates. On the day of transfection, discard the complete medium and add serum-free medium. Add lipo8000 and siRNA according to the manufacturer's instructions. After 6 hours of transfection discard the serum-free medium and add complete medium. Cell functional assays can be performed after 24 hours of culture, and protein extraction and RNA extraction can be performed after 48 hours of culture.

### Statistical analysis

Data were evaluated using Mann-Whitney U test or an independent t test. GraphPad Prism 9 (GraphPad prism 9.0.0, USA) was used for the analysis. Statistical significance was set at P < 0.05.

## Results

### High ECM stiffness enhances docetaxel resistance in PCa

**Figure 1:**
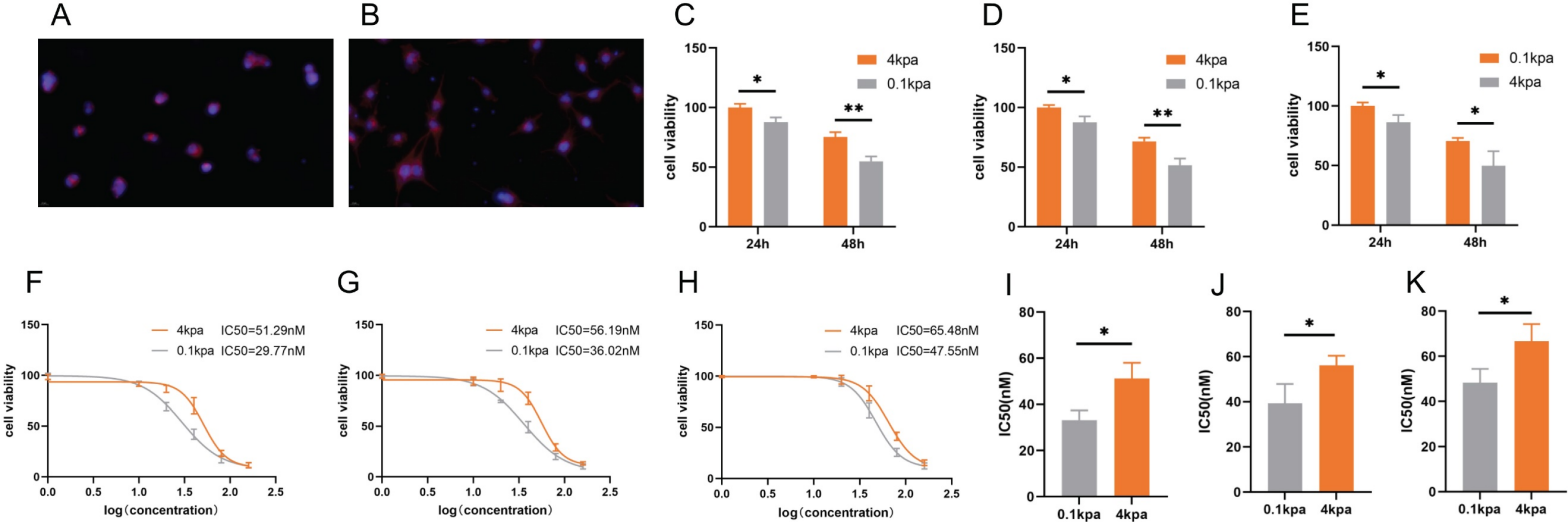
The sensitivity of PC-3, C4-2B and DU145 cells cultured on different stiffness substrates to docetaxel. (A) Representative image of Phalloidin staining of PC-3 cultured on stiff substrates. (B) Representative image of Phalloidin staining of PC-3 cultured on soft substrates. (C) CCK-8 assay results of PC-3 cells cultured on stiff and soft substrates after treatment with 10nM docetaxel for 24h and 48h. (D) CCK-8 assay results of C4-2B cells cultured on stiff and soft substrates after treatment with 10nM docetaxel for 24h and 48h. (E) CCK-8 assay results of DU145 cells cultured on stiff and soft substrates after treatment with 10nM docetaxel for 24h and 48h. (F) Representative dose-effect curve and IC_50_ value of docetaxel on PC-3 cells cultured on stiff and soft substrates. (G) Representative dose-effect curve and IC_50_ value of docetaxel on C4-2B cells cultured on stiff and soft substrates. (H) Representative dose-effect curve and IC_50_ value of docetaxel on DU145 cells cultured on stiff and soft substrates. (I) Statistical analysis of IC_50_ value of PC-3 cells cultured on stiff and soft substrates treated with docetaxel. (J) Statistical analysis of IC_50_ value of C4-2B cells cultured on stiff and soft substrates treated with docetaxel. (K) Statistical analysis of IC_50_ value of DU145 cells cultured on stiff and soft substrates treated with docetaxel.

We prepared polyacrylamide gels with varying stiffness and selected gels possessing an elastic modulus of 0.1 kPa to mimic a soft environment and 4 kPa to emulate a stiff environment. Subsequently, PC3 cells cultured on these gels were stained with phalloidin. The staining results indicated that the PC3 cells adhered well to both gel surfaces. Under low stiffness conditions (0.1 kPa), the PC3 cells exhibited convergence and adopted a morphology characterized by small, rounded or polygonal shapes (*Figure 1A*). Conversely, under high stiffness conditions (4 kPa), the PC3 cells were fully spread and displayed an elongated or large polygonal morphology (*Figure 1B*). To examine the impact of ECM stiffness on docetaxel resistance in PCa, we evaluated the cell viability of PC-3, C4-2B and DU145 cells cultured on substrates with varying stiffness after 24 h and 48 h of docetaxel treatment (*Figure 1C-E*). CCK-8 assays revealed that cells cultured on stiff substrates exhibited lower susceptibility to docetaxel compared to those on soft substrates. Consistently, the IC_50_ values for PC-3, C4-2B and DU145 cells were higher on stiff substrates than on soft ones (*Figure 1F-K*).

### High ECM stiffness suppresses docetaxel-induced apoptosis

**Figure 2:**
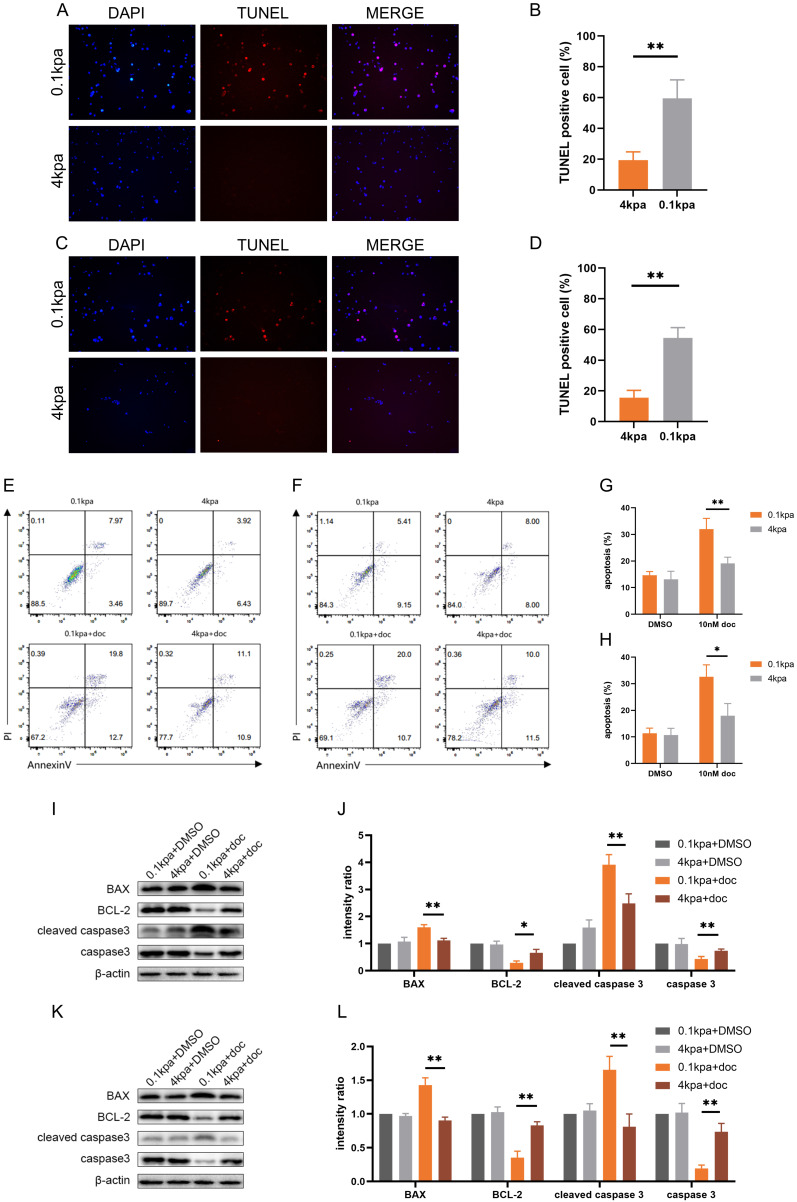
Apoptosis levels of PC-3 cells and C4-2B cells cultured on different stiffness substrates after docetaxel treatment. (A) TUNEL staining of PC-3 cells after treatment with 10nM docetaxel for 24h cultured on stiff and soft substrates. (B) Statistical analysis of the proportion of TUNEL positive cells in PC-3 cells. (C) TUNEL staining of C4-2B cells after treatment with 10nM docetaxel for 24h cultured on stiff and soft substrates. (D) Statistical analysis of the proportion of TUNEL positive cells in C4-2B cells. (E) Flow cytometry of PC-3 cells treated with 10 nM docetaxel for 24 hours cultured on stiff and soft substrates. (F) Statistical analysis of apoptosis rate of PC-3 cells. (G) Flow cytometry of C4-2B cells treated with 10 nM docetaxel for 24 hours cultured on stiff and soft substrates. (H) Statistical analysis of apoptosis rate of C4-2B cells. (I) Expression of apoptosis-related proteins in PC-3 cells treated with 10 nM docetaxel for 24 hours cultured on stiff and soft substrates. (J) Statistical analysis of the expression of apoptosis-related protein in PC-3 cells. (K) Expression of apoptosis-related proteins in C4-2B cells treated with 10 nM docetaxel for 24 hours cultured on stiff and soft substrates. (L) Statistical analysis of the expression of apoptosis-related protein in C4-2B cells.

Docetaxel exerts its therapeutic effect primarily by inducing apoptosis in cancer cells^28^. Following treatment with 10 nM docetaxel for 48 h, TUNEL staining of PC-3 and C4-2B cells cultured on substrates of different stiffness demonstrated that the number of TUNEL-positive cells was significantly lower on stiff substrates compared to soft ones (*Figure 2A-D*). Flow cytometry analysis further confirmed that the apoptosis rate of PC-3 and C4-2B cells was significantly reduced on stiff substrates (*Figure 2E-H*). Western blot results also indicated that the level of apoptosis was markedly downregulated in the high stiffness group compared to the low stiffness group after docetaxel treatment (*Figure 2I-L*).

### Integrins mediate the process of high ECM stiffness promoting docetaxel resistance

**Figure 3:**
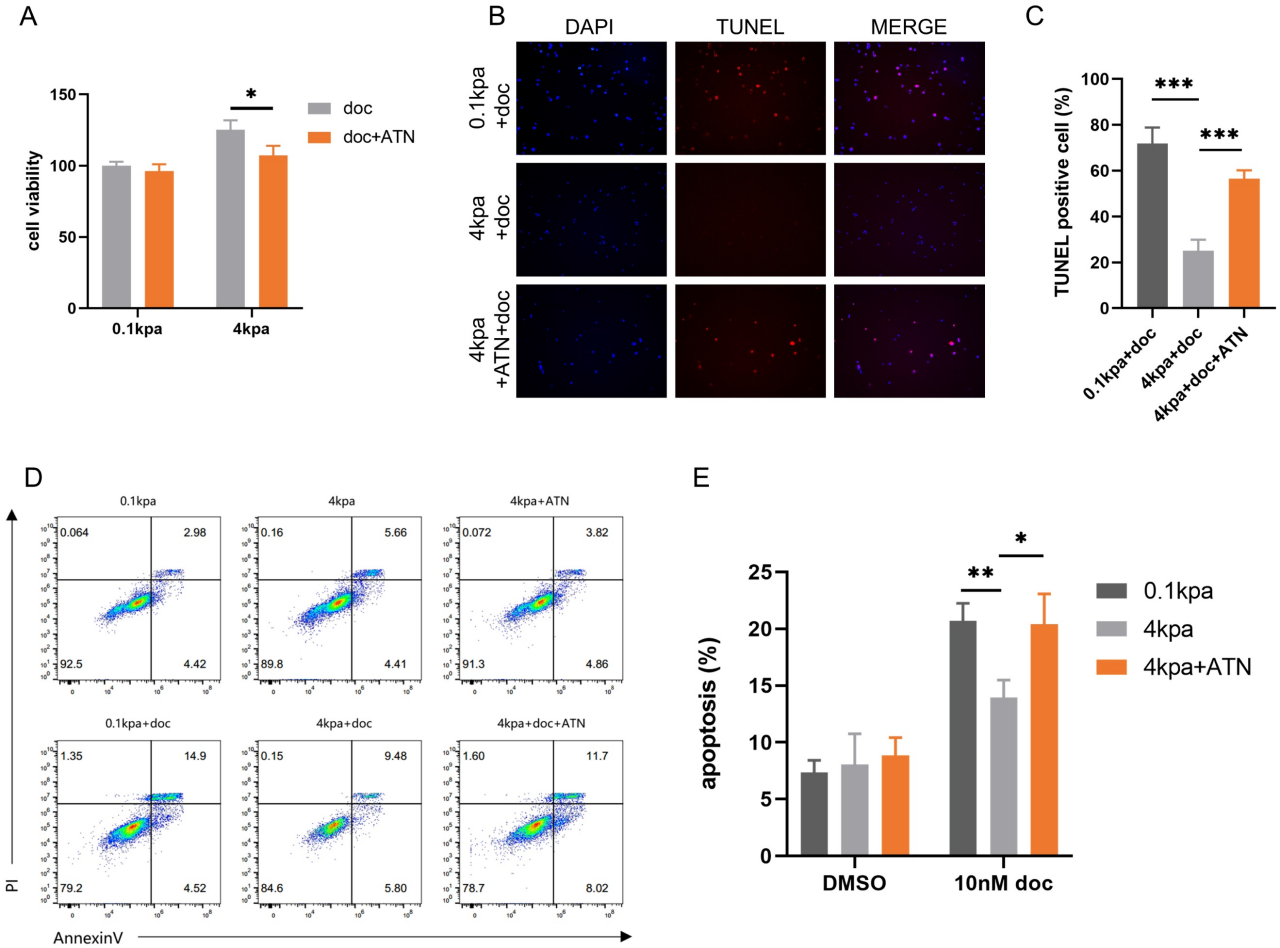
ATN-161 inhibits the anti-apoptotic activity of PC-3 cells mediated by stiff substrates. (A) CCK-8 assays results of PC-3 cells cultured on stiff and soft substrates treated with 10nM docetaxel alone or combined with ATN-161 for 24h. (B) TUNEL staining of PC-3 cells cultured on stiff and soft substrates treated with 10nM docetaxel alone or combined with ATN-161 for 24h. (C) Statistical analysis of the proportion of TUNEL positive cells in PC-3 cells. (D) Flow cytometry of PC-3 cells cultured on stiff and soft substrates treated with 10nM docetaxel alone or combined with ATN-161 for 24h. (E) Statistical analysis of apoptosis rate of PC-3 cells.

Integrins serve as a bridge between extracellular mechanical forces and intracellular structures^29^. Specifically, α5β1 integrin has been implicated in prostate cancer invasion and metastasis^30^, ^31^, as well as in mediating drug resistance across various cancer types^32^, ^33^. Based on these findings, we hypothesized that α5β1 integrin plays a pivotal role in promoting docetaxel resistance in prostate cancer under high matrix stiffness conditions. CCK-8 assays indicated that the α5β1 integrin inhibitor ATN-161 reversed the decreased sensitivity of PC-3 cells to docetaxel (*Figure 3A*). TUNEL staining showed that ATN-161 reversed the decrease in TUNEL-positive cells in PC-3 cells mediated by stiff substrates (*Figure 3B-C*). Flow cytometry analysis further demonstrated that the integrin inhibitor reversed the decrease in apoptosis rates in PC-3 cells (*Figure 3D-E*). These findings suggest that the process by which high ECM stiffness promotes docetaxel resistance in PCa is mediated through an integrin-dependent mechanotransduction pathway.

### Effects of ECM stiffness on the transcriptome of PC-3 cells

**Figure 4:**
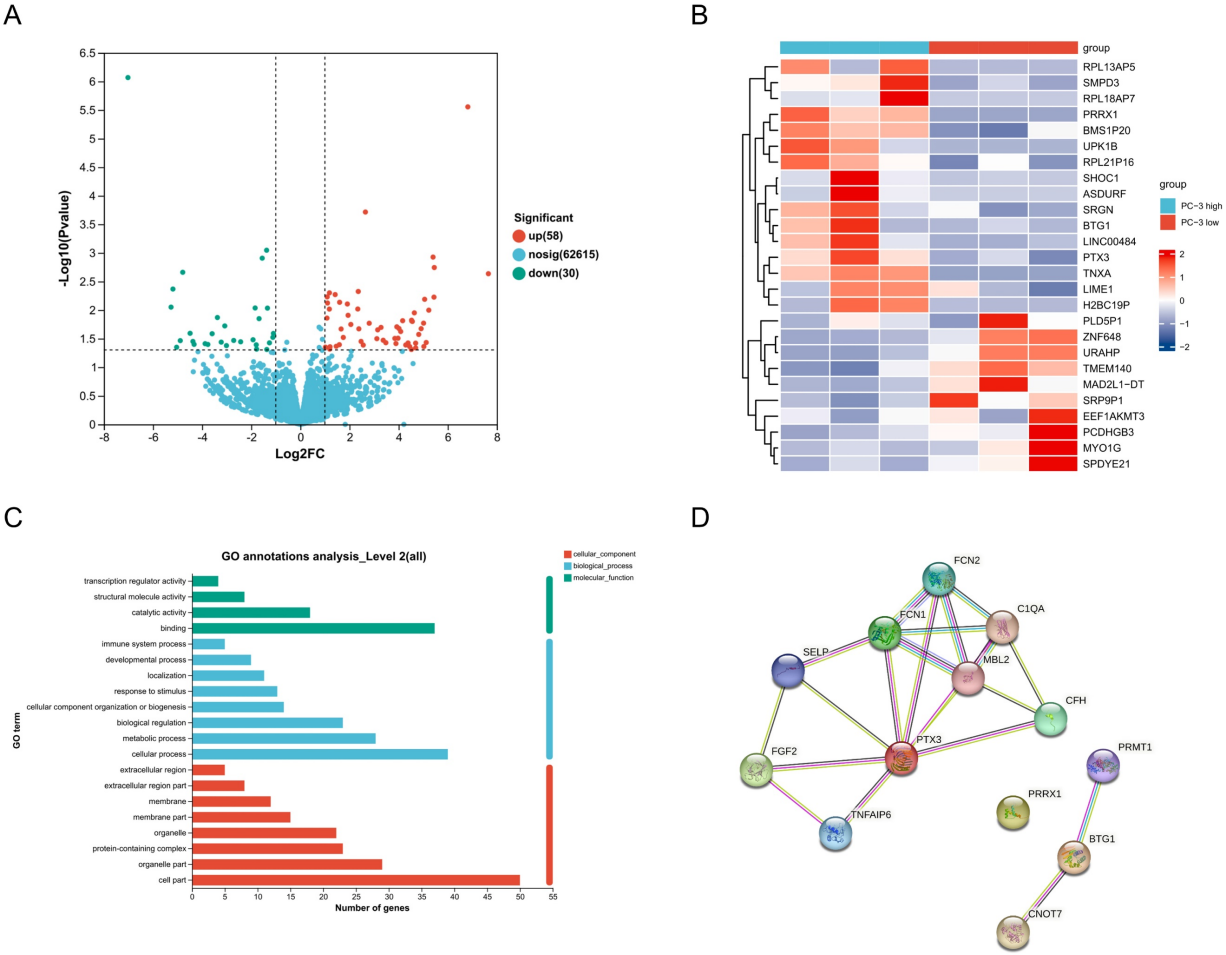
Effects of ECM Stiffness on the Transcriptome of PC-3 Cells. (A) Statistics of gene expression differences in PC-3 cells cultured on different stiffness substrates. (B) Top 26 genes with the most significant changes in expression in PC-3 cells cultured on different stiffness substrates. (C) The GO analysis of differentially expressed genes. (D) Protein-protein interaction network of differentially expressed genes.

Transcriptome sequencing of PC-3 cells under different stiffness conditions revealed that 58 genes were upregulated and 30 genes were downregulated in the high stiffness group compared to the low stiffness group (*Figure 4A*). Heatmap analysis of the top 26 most significantly differentially expressed genes is shown in *Figure 4B*. GO analysis indicated that these differentially expressed genes are primarily involved in biological processes such as immune response, response to stimulation, cellular composition, biological regulation, and metabolic processes (*Figure 4C*). Using the STRING network database, we constructed a protein-protein interaction network for differentially expressed genes under different stiffness conditions (*Figure 4D*), revealing interactions among 13 genes. Among these, PRRX1 was identified as a potential key gene.

### PRRX1 is a critical factor in high ECM stiffness-induced docetaxel resistance in PCa

**Figure 5:**
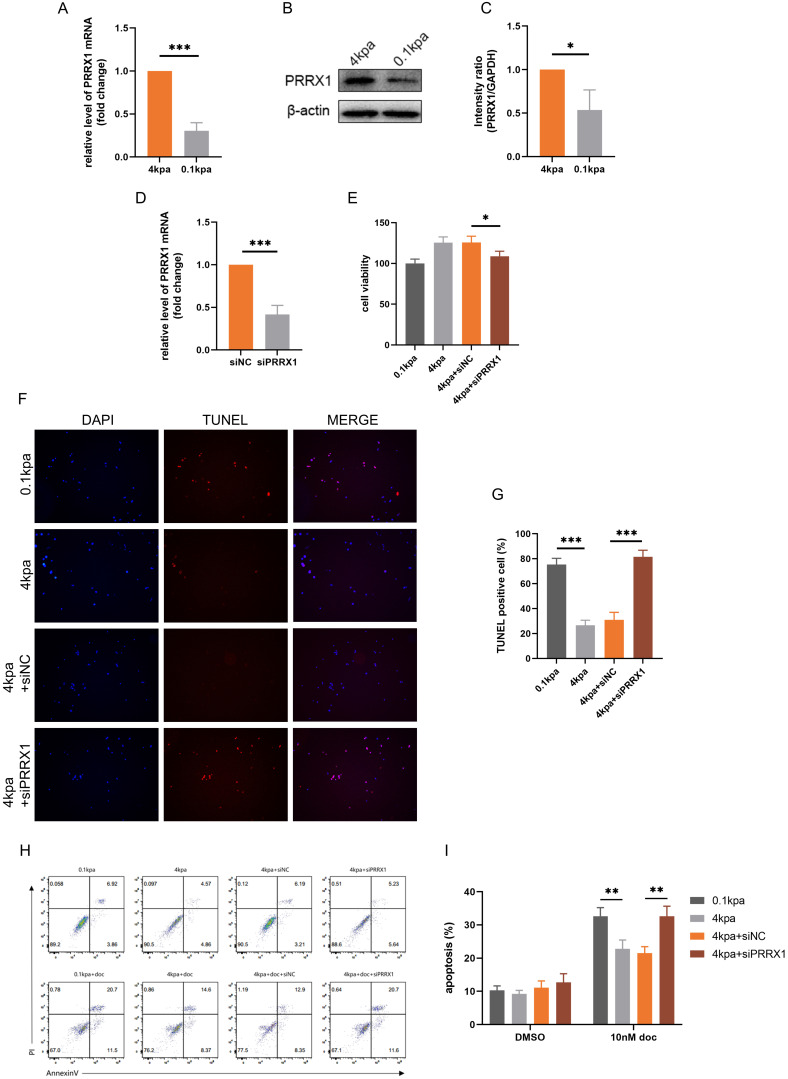
Knockdown of PRRX1 inhibits the anti-apoptotic activity of PC-3 cells mediated by stiff substrates. (A) PRRX1 mRNA levels in PC-3 cells cultured on different stiffness substrates. (B) PRRX1 protein expression in PC-3 cells cultured on different stiffness substrates. (C) Statistical analysis of PRRX1 protein expression in PC-3 cells. (D) PRRX1 expression in PC-3 cells treated with siNC and siPRRX1. (E) CCK-8 assays results of PC-3 cells with or without PRRX1 knockdown, cultured on stiff and soft substrates after 10nM docetaxel treatment. (F) TUNEL staining of PC-3 cells with or without PRRX1 knockdown, cultured on stiff and soft substrates after 10nM docetaxel treatment. (G) Statistical analysis of the proportion of TUNEL positive cells in PC-3 cells. (H) Flow cytometry of PC-3 cells with or without PRRX1 knockdown, cultured on stiff and soft substrates after 10nM docetaxel treatment. (I) Statistical analysis of apoptosis rate of PC-3 cells.

Previous studies have linked PRRX1 to EMT and potential involvement in multidrug resistance in tumors^34^. qPCR and Western blot analyses showed that PRRX1 expression was higher in the high stiffness group compared to the low stiffness group (*Figure 5A-C*). We knocked down PRRX1 expression in PC-3 cells by transfecting siPRRX1 and confirmed the knockdown (*Figure 5D*). CCK-8 assays indicated that knockdown of PRRX1 expression reversed the decreased sensitivity of PC-3 cells to docetaxel (*Figure 5E*). TUNEL staining and flow cytometry revealed that knockdown of PRRX1 expression reversed docetaxel resistance induced by stiff substrate (*Figures 5F-I*). These experimental results demonstrate that PRRX1 plays a pivotal role in the process by which high ECM stiffness promotes docetaxel resistance in PCa.

## Discussion

In this study, we observed that elevated ECM stiffness promotes docetaxel resistance in PCa by inhibiting apoptosis, with PRRX1 identified as a pivotal gene in this process.

Polyacrylamide gel, serving as a straightforward two-dimensional material devoid of confounding variables, offer a valuable tool to investigate the impact of substrate stiffness on cancer cell responses to various stimuli^35^. Its chemical simplicity, ease of fabrication, and cost-effectiveness facilitate their preparation, rendering cell culture on polyacrylamide gels a crucial method for studying ECM stiffness in tumor microenvironments^36^. Consequently, polyacrylamide gel was selected in this study to mimic various ECM stiffness levels, laying the groundwork for subsequent investigations into the effects of ECM stiffness on docetaxel sensitivity in PCa.

Docetaxel, a second-generation paclitaxel-based chemotherapeutic agent, is the standard of care for advanced PCa^37^, necessitating a thorough understanding of docetaxel resistance mechanisms. While the tumor microenvironment has been implicated in docetaxel resistance, the specific role of ECM stiffness in PCa resistance to docetaxel remains unexplored. Our findings reveal that PCa cells cultivated on stiff substrates exhibit a propensity for docetaxel resistance, suggesting potential avenues for addressing this resistance.

Integrins, as key transmembrane adhesion molecules, play a crucial role in regulating cellular responses to mechanical forces^38^. They facilitate the transduction of mechanical forces into cells, thereby modulating critical cellular functions such as migration, invasion, growth, and proliferation^39^. Given the significance of integrins in mechanotransduction pathways, research focusing on integrin inhibitors as antitumor agents has surged^40^, ^41^. Our study confirms that ECM stiffness modulates docetaxel sensitivity in PCa through an integrin-dependent mechanotransduction pathway, highlighting integrins as a promising target for overcoming docetaxel resistance in PCa.

PRRX1, a transcription factor located on chromosome 1q24, is abnormally expressed in various diseases and is implicated in tumor metastasis^42^. It has been shown to mediate drug resistance in tumors by activating specific signaling pathways. In colorectal cancer, for instance, high PRRX1 expression correlates with metastasis, chemoresistance, and poor prognosis^43^. PRRX1 upregulation promotes proliferation, stemness, and chemoresistance in colorectal cancer cells by activating the interleukin-6 (IL-6)/JAK2/STAT3 axis, with IL-6 inhibition reversing its effects on stemness and chemoresistance. Additionally, SPOCK1 upregulation in colorectal cancer cells enhances 5-fluorouracil resistance by regulating PRRX1 expression and downstream apoptosis signaling^44^. In breast cancer, PRRX1 overexpression may induce multidrug resistance through the PTEN/PI3K/Akt signaling pathway and promote tamoxifen resistance in MCF-7 cells via EMT induction^45^. Using RNA-seq and bioinformatics approaches, we demonstrated that high ECM stiffness upregulates PRRX1 expression in PC-3 cells, thereby fostering docetaxel resistance.

This study has certain limitations. For example, future research could integrate molecular mechanisms from basic research with clinical sample variations by collecting docetaxel-resistant clinical samples. Additionally, animal experiments could be incorporated to investigate the upstream mechanisms of ECM stiffness changes in PCa. In conclusion, our novel finding that increased ECM stiffness mediates docetaxel resistance in PCa, when combined with recent research on antitumor therapies targeting ECM stiffness, holds promise for developing new strategies to treat advanced PCa.

## Conclusion

In conclusion, the current study has demonstrated that ECM stiffness can induce docetaxel resistance in PCa cells via the integrin-associated mechanotransduction pathway. Specifically, an augmentation in ECM stiffness was found to upregulate the expression of PRRX1, ultimately facilitating docetaxel resistance in PCa cells. This investigation has elucidated the molecular mechanism underlying docetaxel resistance in PCa, offering a novel perspective for the development of therapeutic strategies targeting PCa.

## Abbreviations

PCa: prostate cancer
ECM: extracellular matrix
ADT: Androgen deprivation therapy
CRPC: castration resistant prostate cancer
EMT: epithelial-mesenchymal transition
